# Generation of Functional Eyes from Pluripotent Cells

**DOI:** 10.1371/journal.pbio.1000174

**Published:** 2009-08-18

**Authors:** Andrea S. Viczian, Eduardo C. Solessio, Yung Lyou, Michael E. Zuber

**Affiliations:** 1 Department of Ophthalmology, State University of New York (SUNY) Upstate Medical University, Syracuse, New York, United States of America; 2 Department of Biochemistry and Molecular Biology, State University of New York (SUNY) Upstate Medical University, Syracuse, New York, United States of America; Harvard Medical School, United States of America

## Abstract

Pluripotent cells such as embryonic stem (ES) and induced pluripotent stem (iPS) cells are the starting point from which to generate organ specific cell types. For example, converting pluripotent cells to retinal cells could provide an opportunity to treat retinal injuries and degenerations. In this study, we used an in vivo strategy to determine if functional retinas could be generated from a defined population of pluripotent *Xenopus laevis* cells. Animal pole cells isolated from blastula stage embryos are pluripotent. Untreated, these cells formed only epidermis, when transplanted to either the flank or eye field. In contrast, misexpression of seven transcription factors induced the formation of retinal cell types. Induced retinal cells were committed to a retinal lineage as they formed eyes when transplanted to the flanks of developing embryos. When the endogenous eye field was replaced with induced retinal cells, they formed eyes that were molecularly, anatomically, and electrophysiologically similar to normal eyes. Importantly, induced eyes could guide a vision-based behavior. These results suggest the fate of pluripotent cells may be purposely altered to generate multipotent retinal progenitor cells, which differentiate into functional retinal cell classes and form a neural circuitry sufficient for vision.

## Abstract 2

The directed differentiation of pluripotent cells into specific cell-types is a major hurdle in regenerative medicine. This study shows the eye field transcription factor factors can direct pluripotent cells into functioning frog eyes.

## Author Summary

The goal of regenerative medicine is to replace dead or dying cells. Successful cell replacement depends on the ability of donor cells to differentiate into all functional cell types lost in the target organ. Blindness resulting from retinal disease or damage, for example, would require the replacement of as many as seven specialized cell types found in the retina. The most celebrated characteristic of pluripotent cells is their ability to differentiate into any adult cell type. This defining feature, however, presents the challenge of identifying the conditions for their conversion to the cell types needed for tissue repair. We asked if pluripotent cells could be directed to generate all the retinal cell types necessary to form a functional eye in the frog, *Xenopus laevis*. If left untreated, transplanted pluripotent cells only form the epidermal layer of the skin. However, when forced to express the eye field transcription factor (EFTF) genes, the cells differentiate into all seven retinal cell classes and eventually organize themselves into a functioning eye that can detect light and guide tadpoles in a vision-based behavior. Our results demonstrate that pluripotent cells can be purposely altered to generate all the functional retinal cell classes necessary for sight.

## Introduction

Every body organ consists of tissue groups with multiple cell types. Therefore, recovery from organ damage requires the replacement of a variety of distinct cell types. The retina is the light detecting tissue of the eye, and consists of seven major cell classes. All retinal cell classes are generated from a common multipotent retinal progenitor. The conversion of pluripotent cells to retinal progenitors would, in theory, provide a source of all the cell classes necessary for retinal repair.

Pluripotent cells treated with extrinsic factors express retinal markers in vitro and in vivo [1]–[5]. When transplanted to the subretinal space of mice lacking functional photoreceptors, human embryonic stem cells directed toward a retinal lineage integrate into the outer nuclear layer, express photoreceptor markers, and restore a light response as determined by the electroretinogram (ERG) [5]. However, it is not known if any other functional retinal cell classes can be derived from pluripotent cells, or if these cells can form the complex neural network necessary for vision.

Vertebrate retina formation begins in the anterior neural plate in a region called the eye field. A group of transcription factors collectively called the eye field transcription factors or EFTFs (*pax6*, *rx1*, *tbx3* [or *ET*], *nr2e1* [or *tailless*], *six3*, *lhx2*, and *six6* [or *optx2*]) and the neural patterning gene *otx2* are essential for normal eye formation [6]. When overexpressed together in developing *Xenopus* embryos by RNA microinjection, these genes can induce eye-like structures as defined by the expression of markers for some retinal cell classes [7]. The overall aim of this study was to determine if pluripotent cells overexpressing these transcription factors could be intentionally driven toward retinal progenitors that differentiate into multiple retinal cell classes and form a functional retina.

Primitive ectoderm cells isolated from the animal pole of blastula stage embryos are pluripotent. If treated with the appropriate inducer, they can form endodermal, mesodermal, or ectodermal cell types [8]. Here we provide evidence that these pluripotent cells misexpressing the EFTFs differentiate into all retinal cell classes. Using both the ERG and a vision-based behavioral assay, we found that eyes generated from EFTF-expressing pluripotent cells are molecularly and functionally similar to the normal eye. These results suggest that by using the correct combination of gene products, it may be possible to reprogram more readily available pluripotent cell types to a multipotent retinal cell lineage useful in healing damaged or diseased retinas.

## Results

### EFTFs Induce an Eye Field-Like Transcriptional Profile in Pluripotent Cells

**Figure 1:**
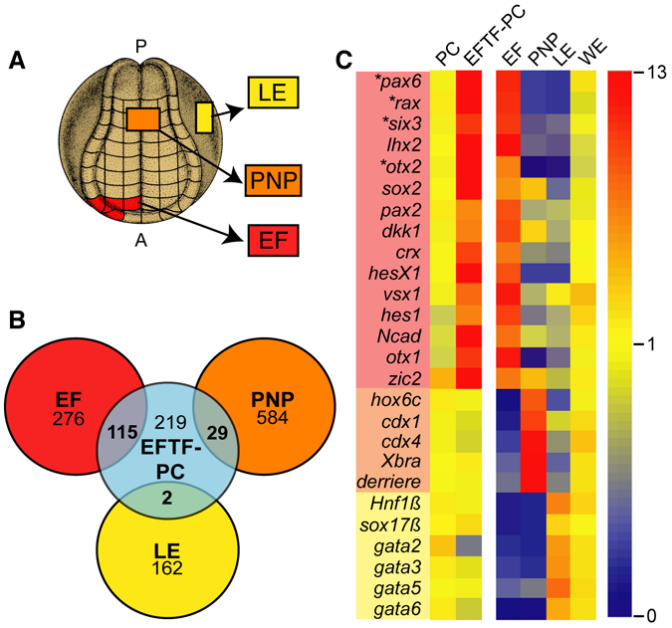
EFTFs induce an eye field-like transcriptional profile. (A) Location of the eye field (EF), PNP, and LE tissue isolated from stage 15 *Xenopus* embryos for microarray analysis. (B) Venn diagram identifying the number of genes induced 2-fold or greater in EFTF-expressing pluripotent cells (EFTF-PC, blue) and enriched 2-fold or greater in eye field (red), PNP (orange), or LE (yellow). (C) Relative expression levels of transcripts required for eye formation (boxed in pink; also see Table S1). Known markers of PNP (boxed in orange) and LE (boxed in yellow) are included for comparison (also see Table S2). Microarray data sets for pluripotent cells (PC), EFTF-PC, EF, PNP, LE, and whole embryos (WE) are shown. High and low expression is indicated in red and blue, respectively. The probe sets detecting these transcripts do not hybridize to the injected RNA. Consequently, the EFTF induction detected here is transcription from the endogenous genes (*pax6*, *rx/rax*, *six3*, and *otx2*; noted by asterisk).

Coordinated misexpression of the EFTFs (*pax6*, *tbx3*, *rx1*, *nr2e1*, *six3*, and *six6*) and *otx2* in developing embryos results in formation of ectopic eye tissue [7]. One interpretation of this result is that EFTFs induce an eye field-like fate in pluripotent cells. This interpretation predicts that transcripts expressed in the eye field should also be induced in EFTF-expressing pluripotent cells. Therefore, we used microarray analysis to perform pairwise comparisons of the transcriptional profile of EFTF-expressing pluripotent cells to three regions of the *Xenopus* embryo: the eye field (EF) including underlying mesendoderm, posterior neural plate (PNP) and the non-neural, flank, lateral endoderm (LE, Figure 1A).

Of approximately 14,400 transcripts evaluated by the Affymetrix GeneChip *X. laevis* Genome Array, 365 were induced greater than 2-fold in EFTF-expressing pluripotent cells. Of the 365 EFTF-induced transcripts, 31.5%, 8%, and 0.5% were uniquely shared with EF, PNP, or LE, respectively (Figure 1B). We used an unsupervised hierarchical clustering algorithm to compare the transcriptional profiles of all samples. This analysis compares the relative change of all probe sets on the array and unbiasedly groups the four tissue samples based on their similarity [9]. These results indicate the eye field and EFTF-expressing pluripotent cells are more closely related to each other than to either the PNP or LE tissues (Figure S1). Importantly, 25 of the 115 transcripts, shared by EFTF-expressing pluripotent cells and the EF, encode for 15 genes that are both expressed in retinal stem/progenitor cells and required for normal eye formation in frogs, fish, mice, or humans (Figure 1C; Table S1). The expression levels for 13 of these transcripts were not significantly different when comparing eye field and EFTF-expressing pluripotent cells (*p*<0.05 considered significant). By comparison, only two of the 15 genes were not significantly different when comparing the eye field to PNP or LE data sets (Table S1). These results suggest EFTF-expressing pluripotent cells share a common transcriptional profile with the eye field, and are consistent with the hypothesis that the EFTFs direct nonretinal pluripotent cells to an eye field-like retinal cell lineage.

### EFTF-Expressing Pluripotent Cells Are Directed to a Retinal Lineage

**Figure 2:**
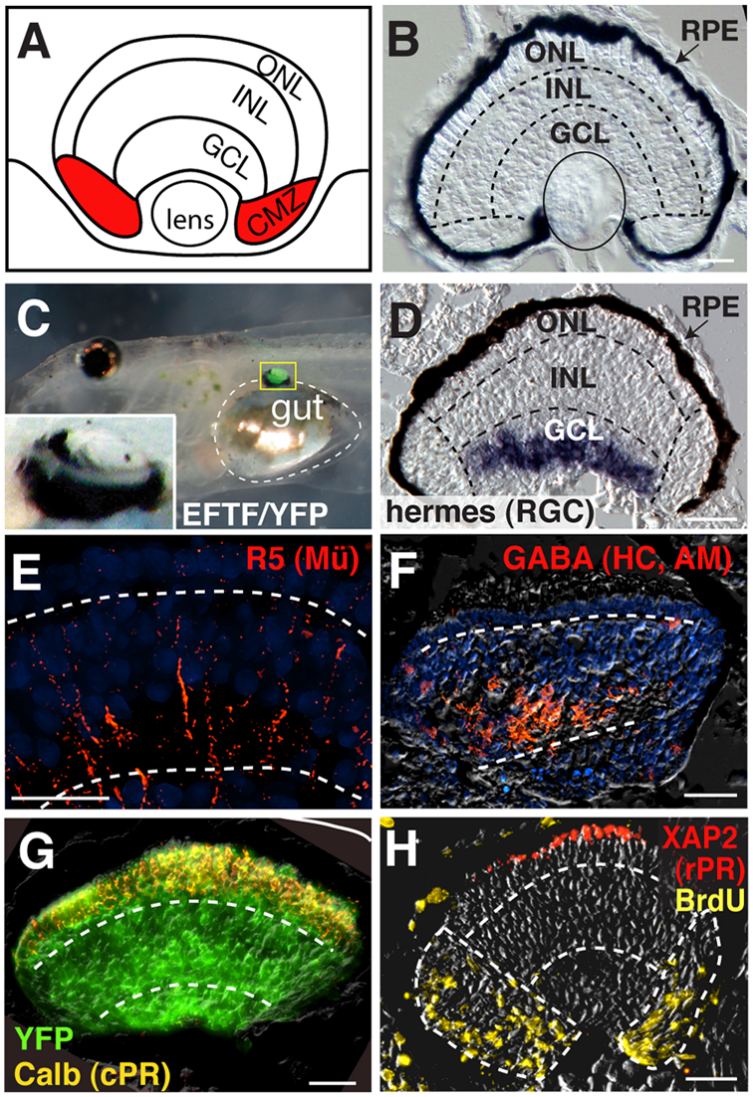
EFTF-expressing pluripotent cells form eye-like structures in tadpole flank. (A) Schematic illustrating the expected retinal layers of the eye including the outer nuclear (ONL), inner nuclear (INL), and ganglion cell (GCL) layers. The mitotically active CMZ is indicated in red. (B) Transverse section of a stage 41 wild-type retina. Dashed lines mark the location of the plexiform layers. The eye is oriented so that dorsal is to the left. (C–G) Flank eyes expressed YFP throughout, contained an RPE and a trilayered morphology with putative inner and outer plexiform layers (dashed lines in D–H). Markers were used to detect retinal cell classes. (C) Whole tadpole bright field image overlayed with fluorescent image, showing flank eye (yellow box) on top of gut (dashed line). Inlay is a magnified view of the boxed flank eye. (D) Cryostat section (12 µm) shows cells labeled for the retinal ganglion cell (RGC) marker, *hermes*, by in situ hybridization. (E) R5 antibody labels Müller glia (Mü; red), which extend processes the breadth of the flank retina. (F) GABA labels horizontal and amacrine cells (HC, AM; red) of the INL. (G) Cells expressing YFP (green) co-express the cone photoreceptor (cPR) marker Calbindin (yellow). (H) Cells expressing the rod photoreceptor marker XAP2 (rPR; red); BrdU-immunoreactivity (yellow) identifies mitotically active cells in the periphery of the same flank retina. Scale bars, 25 µm.

The EFTFs may transiently alter the pattern of gene expression in pluripotent cells, yet fail to stably direct cells toward a retinal cell fate. To test this possibility, we transplanted EFTF-expressing pluripotent cells to the flank of *X. laevis* embryos. To ensure that donor and host tissues could be easily distinguished, we generated EFTF-expressing cells from transgenic embryos constitutively expressing a variant of yellow fluorescent protein (Venus YFP) [10]. Transplanted control pluripotent cells isolated from Venus YFP embryos formed only sheets of epidermis (number of animals or eyes [N] = 108; unpublished data). In contrast, EFTF-expressing pluripotent cells formed YFP-expressing, pigmented spheroids in 23% of transplants (N = 566). We compared the morphology of the induced tissues to the normal eye (Figure 2A and 2B). Thirty-one transplants (24% of pigmented spheroids) had an eye-like morphology consisting of retinal pigment epithelium (RPE) and the trilayered cup structure of a normal retina (Figure 2C and 2D). We used immunofluorescence and in situ hybridization to detect specific retinal cell classes. All 31 expressed YFP throughout and molecular markers for two or more retinal cell classes. Markers used included Islet-1 and *hermes* for retinal ganglion cells, Tyrosine Hydroxylase for amacrine cells, R5 for Müller glia, XAP2 for rod photoreceptors, and Calbindin for cone photoreceptors (Figure 2D, 2E, 2G, and 2H; Tables S3 and S4). The inner nuclear layers of flank retinas were also strongly labeled for gamma-aminobutyric acid (GABA), which stains horizontal, and a subset of amacrine cells, and Calretinin, which predominantly labels bipolar cells but also subsets of amacrine and retinal ganglion cells (Figure 2F; Tables S3 and S4). These results suggest that EFTF-expressing cells, like bona fide eye field cells, are determined, forming retinal cells and even eye-like structures, when transplanted to another region of the embryo.

In addition to differentiated retinal cells, the amphibian eye contains a population of self-renewing retinal stem cells located in the retinal periphery in a region called the ciliary marginal zone or CMZ. Retinal stem cells are derived from the embryonic eye field and are the source of new retinal cells throughout the life of the animal [11],[12]. To determine if mitotic cells were present in the periphery of flank eyes, tadpoles were briefly placed in a solution containing 5-bromo-2-deoxyuridine (BrdU). BrdU immunoreactivity was detected in all retinas tested (Figure 2H; Table S4). To determine if mitotic cells were enriched in the peripheral retina, we determined the relative proportion of BrdU-positive cells in the peripheral and central retina. We found that 12.5% of peripheral cells yet only 0.6% of central cells were BrdU-labeled in flank retinas (2,063 cells from ten flank retinas were counted, *p*<0.001). We observed similar percentages in wild-type retinas, which contain 18.6% and 0.12% BrdU-labeled peripheral and central cells, respectively (2,672 cells counted in ten control retinas). Based on a Student's paired *t*-test, there was no statistically significant difference in the percentage of BrdU-labeled cells when control and flank eyes were compared (*p*>0.1).

Despite the remarkable ability of EFTF-expressing pluripotent cells to form eye-like structures on the tadpole flank, it was not possible to record ERGs because of the small size of the induced flank eyes (flank eye volume: 0.007±0.003 mm^3^; control eye volume: 0.021±0.002 mm^3^, at stage 41, *N* = 5). In addition, retinal ganglion cell axons exiting the flank eyes failed to reach their normal tectal targets. When DiI was applied to the RGC layer of induced eyes, labeled processes were observed projecting dorsocaudally along the spinal cord away from the tectum (unpublished data). Similar paths are taken by RGC axons exiting ectopic eyes generated by transplantation of eye primordia [13]–[15]. Misrouting has been attributed to the absence of the directional cues required for normal RGC axon guidance. These observations prompted us to replace the eye field with EFTF-expressing pluripotent cells to ask if the induced retinal cells could generate a normal eye and if induced retinal ganglion cells would extend axons to and synapse with their normal tectal targets.

### EFTF-Expressing Pluripotent Cells Generate Morphologically Normal Eyes

**Figure 3:**
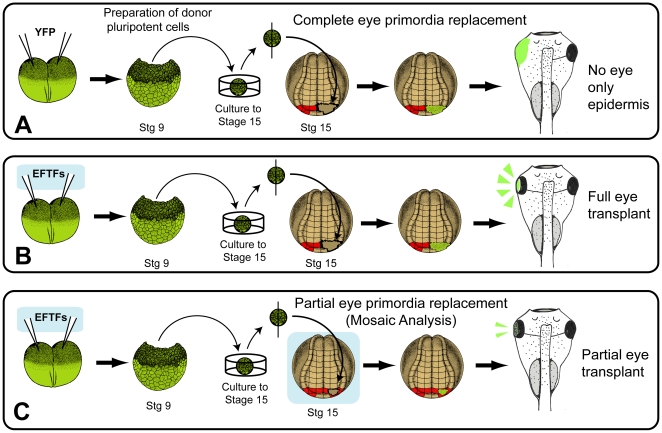
Schematic of the transplant procedure used to assess the fate of EFTF-expressing pluripotent cells. (A) YFP-only, or (B and C) EFTF RNAs were injected into both blastomeres of two-cell stage embryos from CAG-Venus YFP transgenic *X. laevis*. At stage 9, pluripotent cells were removed and cultured to the equivalent of stage 15. For experiments requiring complete eye field replacement (A and B), one half of the eye field was surgically removed from wild-type, stage 15 (host) embryos, and replaced with the donor cells from one half of an explant. (C) To create mosaic eyes, approximately one-half of one eye field was removed and a size-matched fragment of donor tissue was grafted to the host eye field. The location of the eye field is shaded red. Schematic embryos adapted from Nieuwkoop and Faber and Eagleson and Harris [38],[42].

*Xenopus* embryos from which an eye field has been removed survive, develop normally, but lack an eye on the operated side. We grafted donor (YFP-only or EFTF/YFP-expressing) pluripotent cells to host embryos from which one of the two eye fields had been removed (Figure 3A and 3B). When cultured in isolation, pluripotent cells isolated from blastula stage embryos autonomously form epidermis, loosing competence to form other structures. We reasoned, however, if cells were successfully reprogrammed to multipotent retinal cells, and grafted to the anterior neural plate, an eye should form.

**Figure 4:**
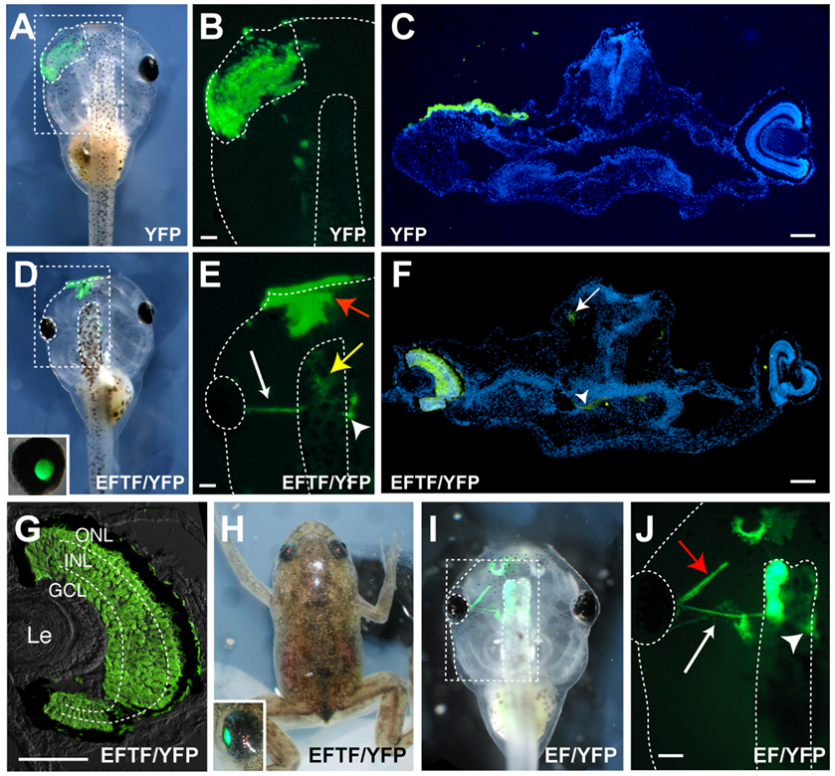
EFTF-expressing pluripotent cells form morphologically normal eyes. (A–C) Control YFP-expressing pluripotent cells (YFP) form only epidermis when grafted to embryos after removal of the endogenous (left) eye field. Bright field and fluorescent images show the location of transplanted cells. (D–H) In contrast, EFTF-expressing pluripotent cells (EFTF/YFP) generate an eye. YFP fluorescence demonstrates the eye originates from donor tissue. Induced eyes grow throughout the life of the animal (E, H), and form the characteristic trilayered structure of a normal retina (G). (E) Retinal ganglion cell axons exit the eye as the optic nerve (white arrow), pass under the brain and out of view then reappear on the contralateral side (arrowhead). Donor cells are sometimes also found in forebrain (yellow arrow) and epidermis (red arrow). (F) YFP expression is detected throughout the eye, in RGC axons (arrowhead) and forebrain (arrow). (I and J) Eye fields (EF/YFP) also generate eyes. In addition to the eye, epidermis, and forebrain, some musculature and olfactory tissue are also derived from donor eye field. White arrow points to ganglion cell axons, while red arrow points to ocular muscle. Arrowhead indicates the location of YFP-positive retinal axons that have projected to the brain. (A–E, I, and J) stage 45 tadpoles; (F and G) stage 46 tadpole; (H) 3-mo-old froglet. In (G), a sectioning artifact introduced a few YFP-negative epidermal cells between the optic cup and lens (Le). Scale bars, 100 µm.

YFP-expressing pluripotent cells grafted in place of the eye field never formed eyes (Figure 4A; N = 79). As expected, these cells generated only epidermis (Figure 4A–4C). In contrast, EFTF-expressing pluripotent cells generated YFP-labeled eyes in 63% of transplants (N = 74). The majority of transplants created mosaic eyes (55%), likely a consequence of the difficulty in surgically removing all traces of the eye field. Nevertheless, 8% of the transplants formed retinas expressing YFP throughout and were therefore completely donor derived. We focused our attention on eyes derived entirely from the EFTF-expressing pluripotent cells (Figure 4D–4H). We observed YFP-labeled processes exiting the induced eye at the optic nerve head that followed the expected trajectory of RGC axons to their tectal targets (Figure 4E). We also observed some YFP-labeled cells in the epidermis, forebrain, and occasionally olfactory bulb (Figure 4E and 4F and unpublished data). Induced eyes contained a lens, RPE, and the trilayered structure of a normal retina (Figure 4G). Interestingly, while the retina and RPE of induced eyes were YFP-labeled, lens cells were most often not, indicating that the lens formed from host tissue (Figure 4G). This result is consistent with previous experiments in *Xenopus* demonstrating the inductive cues and origin of the retinal and lens primordia are distinct [16],[17].

These observations suggest the EFTFs can direct pluripotent cells to an embryonic eye field-like cell fate. If this hypothesis is correct, transplantation of eye fields should yield similar results. Therefore, we transplanted embryonic eye fields from YFP transgenic embryos to wild-type hosts. Embryonic eye fields formed mosaic and complete eyes at frequencies similar to those observed with EFTF-expressing pluripotent cells (mosaic eyes 68%, complete eyes 16%; N = 38; Figure 4I and 4J). These results suggest EFTF-expressing pluripotent cells are directed to an embryonic eye field-like cell fate. Interestingly, in addition to the tissues formed by EFTF-expressing pluripotent cells, transplanted eye fields also formed muscle and head mesenchyme (Figure 4J and unpublished data).

### EFTF-Expressing Pluripotent Cells Generate All Retinal Cell Classes

If the EFTFs induce multipotent retinal progenitor cells, every retinal cell class, including retinal stem cells, should form in induced eyes. To better discern cell classes and determine how well donor cells integrated into the retina we deliberately generated mosaic eyes. EFTF/YFP expressing pluripotent cells were transplanted to embryos from which only one-half of one eye field had been removed (Figure 3C). This allowed us to directly compare the patterns of marker expression in normal and induced retina, and identify cell classes on the basis of their distinctive morphology and location within the eye.

**Figure 5:**
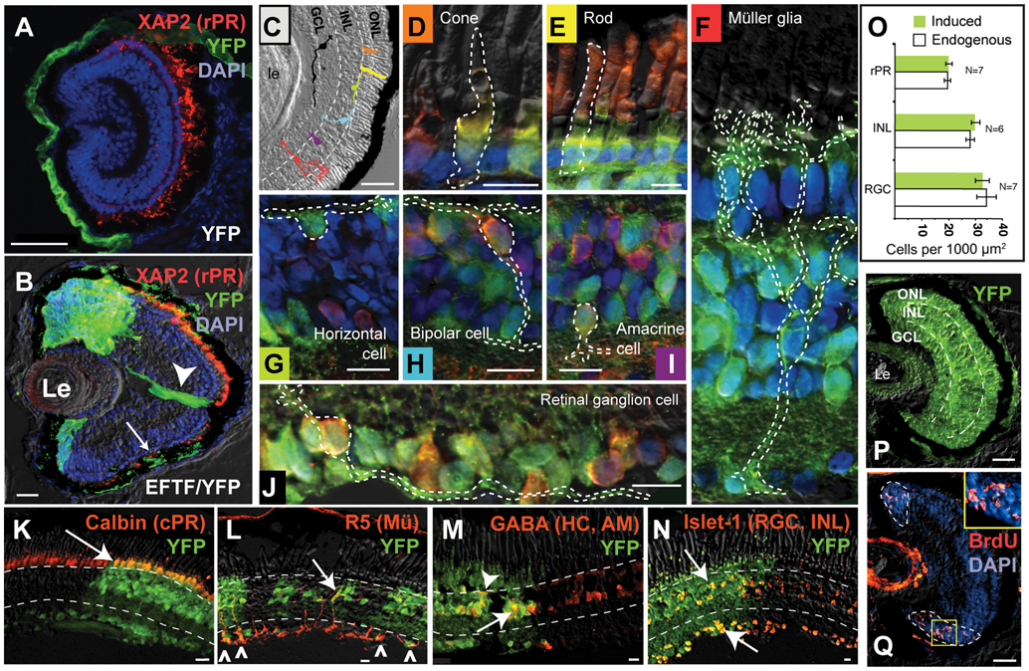
EFTF-expressing pluripotent cells generate all retinal cell classes, RPE, and mitotic cells in the CMZ. (A) Control, pluripotent cells (green) never formed retinal tissue. (B) In contrast, EFTF/YFP-expressing cells generate mosaic retinas with columns of cells spanning the entire retinal width. Retinal ganglion cell axons (arrowhead), RPE (arrow), and rod photoreceptors (stained red for XAP2) are donor derived. (C) Bright field image of a representative retina to illustrate the expected location and morphology of retinal cell classes. The color of cells in (C) is matched to the appropriate cell in (D–J). (D–J) Examples of single YFP-labeled cells identified on the basis of their distinctive morphology (outlined), location within the mosaic retina, and expression of cell class specific markers. Cells are oriented with the RPE side of the retina at the top and lens to the bottom. Z-series images were used to determine the physical boundary of each cell (see Figures S4, S5, S6 for examples). Cones (D) and rods (E) appear greenish/yellow when costained for YFP and Calbindin (red) or XAP2 (red), respectively. XAP2 labels rod outer segments strongly, while YFP is predominantly localized to rod inner segment. Müller glial (F) and horizontal (G) cells express YFP. Bipolar (H), amacrine (I), and retinal ganglion (J) cells double labeled for YFP and Calretinin appear reddish-yellow. (K–N) Expression pattern of cell class markers is continuous through host and induced regions of mosaic retinas (see also Figure S2). Mosaic retinas stained for Calbindin (cones, K), R5 (Müller glia, L), GABA (horizontal and amacrine, M), and Islet-1 (retinal ganglion and inner nuclear layer cells, N). Host (YFP) cells appear red when stained for cell class specific markers, while double-labeled, donor-derived cells (arrows, arrowheads, and carrots) appear yellowish-red. (O) Comparison of RGC, inner nuclear layer, and rod photoreceptor cell densities in endogenous and induced regions of mosaic retinas. Graphs show mean±SEM, *n*>500 cells for each cell class. (P and Q) BrdU-labeled (red) cells detected in the CMZ (outlined in Q) of induced eyes. Sections in (A, B, P, and Q), (C–J), and (K–N) are of stage 46, 50, and 54 tadpoles, respectively. Scale bars, (A–C) = 50 µm; (D–N) = 10 µm; (P, Q) = 25 µm.

Control, YFP-only expressing pluripotent cells never generated mosaic retinas (Figures 5A and S2A; N = 57). This demonstrates that even though the anterior neural plate is essential for proper eye morphogenesis, cultured pluripotent cells were not directed to a retinal fate—even when grafted directly into the eye field. In striking contrast, cultured EFTF-expressing pluripotent cells generated the seven classes of differentiated retinal cells observed in the normal retina. Retinal cell classes were identified based on their morphology, location within the retina, and using cell class specific markers (Figures 5 and S2, S3, S4, S5, S6). RGCs lie on the vitreal surface of the retina adjacent to the lens. RGC axons are the only neural processes that leave the retina. Consistent with the presence of donor-derived RGCs, YFP-labeled processes were detected exiting the eye at the optic nerve head (Figures 4E, 5B, and S2B). All other cell classes were similarly identified on the basis of their morphology and retinal location (Figures 5C–5J, S2, S3, S4, S5, S6).

Molecular markers for retinal ganglion, amacrine, bipolar, horizontal, Müller glia, and rod and cone photoreceptor cells (Table S3) identified these cell types (Figures 5B, 5D–5N, and S2, S3, S4, S5, S6). The expression patterns of cell class-specific markers in mosaic retinas appeared the same in host and donor derived regions suggesting that donor cells generated all retinal cell classes in approximately normal ratios (Figures 5K–5N and S2). Consistent with this idea, we observed no significant difference in retinal ganglion, inner nuclear layer, or rod photoreceptor cell density when endogenous and induced regions of mosaic retinas were compared (Figure 5O). We did not stain every mosaic retina for all seven retinal cell classes. However, we typically stained each mosaic retina for three or four cell class-specific markers and always detected cells expressing every marker tested (three of three or four of four). In addition, every mosaic retina contained columns of YFP-positive cells that spanned the entire width of the retina—from RPE to RGCs (see Figures 5B, 5K–5N, and S2B–S2J for examples). The layering within these columns was indistinguishable from the adjacent, control retina and contained cells with the appropriate morphology for their nuclear layer (rods and cones in the ONL, bipolar, amacrine, horizontal, and Müller glia in the INL, and RGCs in the GCL). These results suggest that EFTF-expressing cells are multipotent, as they differentiate into the seven retinal cell classes of the mature retina.

We also cultured animals in BrdU and used immunocytochemistry to detect mitotically active cells in mature retinas generated from EFTF-expressing cells. BrdU immunoreactivity was detected in the dorsal and ventral, donor-derived CMZ of every animal tested (Figure 5P and 5Q; N = 43). BrdU immunoreactivity is not conclusive evidence for the presence of retinal stem cells. However, when coupled with the observation that induced eyes continued to grow throughout the life of the animals (Figure 4D and 4H), these results suggest EFTF-induced eyes contain a self-renewing population of retinal cells in the retinal stem cell niche of the CMZ.

### Induced Retinal Cells Form Functional Eyes

**Figure 6:**
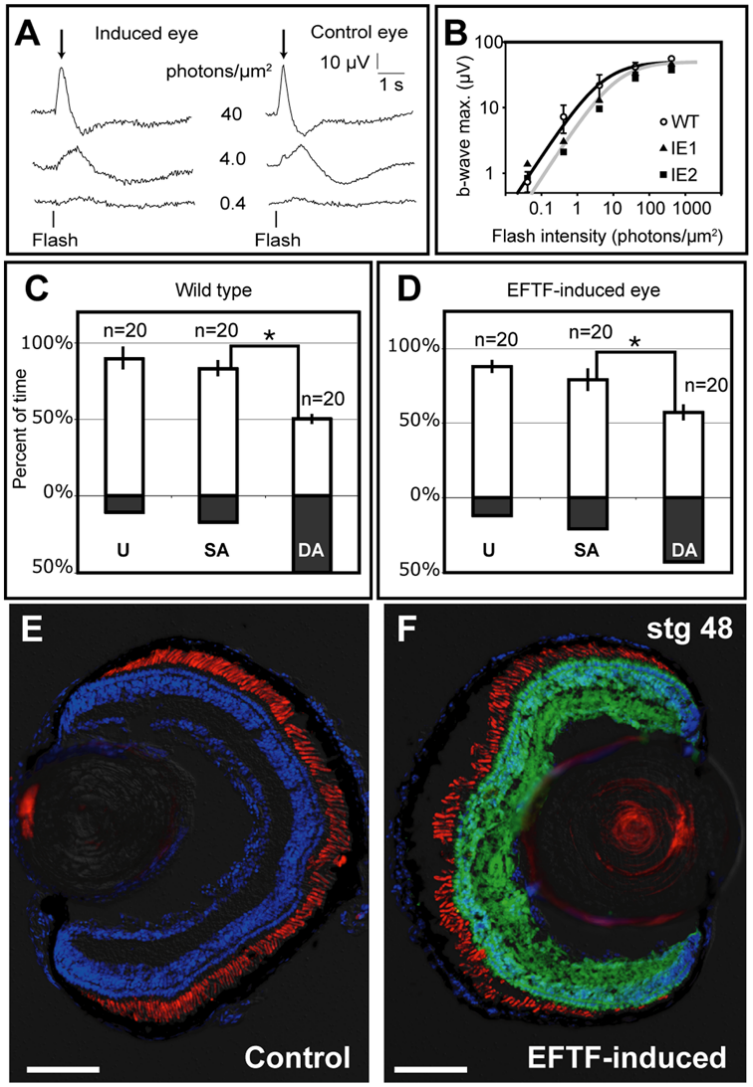
Eyes generated from induced retinal cells are functional. (A and B) ERG responses of control (WT) and induced eyes (IE) are similar. (A) Representative ERG response of the induced and control eye recorded from the same animal. Traces represent the average of eight or more responses. Flash was 520 nm, 20 ms in duration. Light intensity is indicated. (B) The b-wave magnitudes of two induced eyes (IE1, IE2) increase with flash intensity. Open symbols represent the average of eight control animals. Plot shows mean±standard deviation. Averages are fit with a Michaelis-Menten function. (C and D) Background color preference histograms of control (C) and experimental (D) animals. Tadpoles were tested before (unoperated, U), and following single (SA) and double (DA) retinal axotomy. White and black histogram bars indicate average percent time spent on white or black side of test tank. Graphs show mean±standard error of the mean. *n* = number of 2-min trials. (E and F) Sections of the control (E) and induced (F) eyes of the animal tested in (D). Cell nuclei, rod outer segments, and donor-derived retina are blue (DAPI), red (XAP2), and green (Venus YFP), respectively. Scale bar, 100 µm.

Normal anatomical structure and the expression of cell class-specific markers does not demonstrate normal cellular function and connectivity. Therefore, we next measured the ERG to determine if induced cells could form the intricate neural network necessary to detect and process light stimuli. ERGs recorded from eyes wholly derived from induced retinal cells were similar to those recorded from eyes on the unoperated side of the same animals and eyes of stage-matched wild-type controls (Figure 6A and 6B). ERG responses of control and induced eyes were similar in sensitivity and time course (Figure 6A). Rod and cone photoreceptors in the outer retina transduce light into electrical signals. Bipolar cells relay the visual information from photoreceptors to the inner retina for further processing. Activation of bipolar cells elicits the characteristic b-wave of the ERG (Figure 6A; arrows). In response to green (520 nm), dim flashes (0.4 photons/µm^2^) the b-waves are small and exhibit slow kinetics. In response to brighter flashes, b-waves were progressively larger and faster. Figure 6B shows the amplitude of the b-waves plotted as a function of flash intensity. b-waves increased progressively with intensity, saturating in response to the brightest flashes (>30 photons/µm^2^). The b-waves of the induced eyes were slightly desensitized (approximately 30%) relative to controls, as reflected by the slight shift of the Michaelis-Menten functions fit to the data (Figure 6B). Immunostaining for YFP confirmed the eyes from which ERGs were recorded were completely derived from donor tissue (unpublished data). ERGs not only indicate the presence of mature retinal cells, but also demonstrate that light enters the eye appropriately, phototransduction takes place, synapses form between retinal neurons, and synaptic transmission is functional within the eye.

The ERGs and the presence of optic nerves projecting to the brain (Figure 4E) prompted us to ask if the animals could respond to a visual stimulus using the induced eye. *Xenopus* show a stage-dependent phototropic behavior; premetamorphic tadpoles raised in a 12-h light cycle prefer a white color background [18]. When placed in a half-white/half-black test tank, sighted animals swim to and remain on the white side. In contrast, the same animals blinded by axotomy of the retinal ganglion cell axons from both eyes (double axotomy) show no color preference. We generated eyes in wild-type hosts using EFTF-expressing pluripotent cells from YFP transgenic donors, grew animals to premetamporphic stages, and tested for phototropic behavior using the Background Color Preference Assay [18]. The majority of transplants (12 of 13) generated mosaic eyes (24%–52% of the retina was YFP-labeled). However, a retina of one animal was entirely derived from EFTF-expressing pluripotent cells. An age-matched control and the animal with one induced eye both showed strong white background preference, spending 89%±6% and 88%±4% of swimming time on the white side of the test tank, respectively (Figure 6C and 6D). We then severed the right optic nerve, leaving the optic nerve from the induced eye intact (single axotomy). Single axotomy was also used to blind the right eye of the control animal. No statistical difference in background color preference was detected following single axotomy, suggesting the induced eye could guide behavior. To confirm this conclusion, we next severed the optic nerve from the induced eye. Double axotomy resulted in a significant reduction in the phototropic behavior of both the control and experimental animal. White background preference was reduced with axotomy of the induced eye (79%±6% to 57%±5%, *p* = 0.009) and the age-matched wild-type control (83%±6% to 50%±2%, *p* = 6.1×10^−5^; Figure 6C and 6D). The left eye did not contain transplanted cells (Figure 6E), while the induced retina expressed YFP throughout, confirming that it formed entirely from the transplanted donor cells (Figure 6F). These results suggest that EFTF-expressing pluripotent cells can form eyes with the functional cell classes and circuitry necessary for phototropic behavior.

### The EFTF-Inducer Noggin also Generates Functional Eyes

Noggin strongly induces the expression of the EFTFs in frog primitive ectoderm and human ES cells [3],[7]. In *Xenopus*, Noggin can direct the progeny of early blastomeres, not normally destined to contribute to the eyes, to a retinal fate [19],[20]. Human ES cells treated with Noggin, Dickkopf-1, and Insulin-like Growth Factor-1 proteins differentiate into functional photoreceptors [5]. Given our ability to test retinal function in the *Xenopus* system, we wondered if mammalian Noggin could mimic the effect of the EFTFs and generate functional eyes.

**Figure 7:**
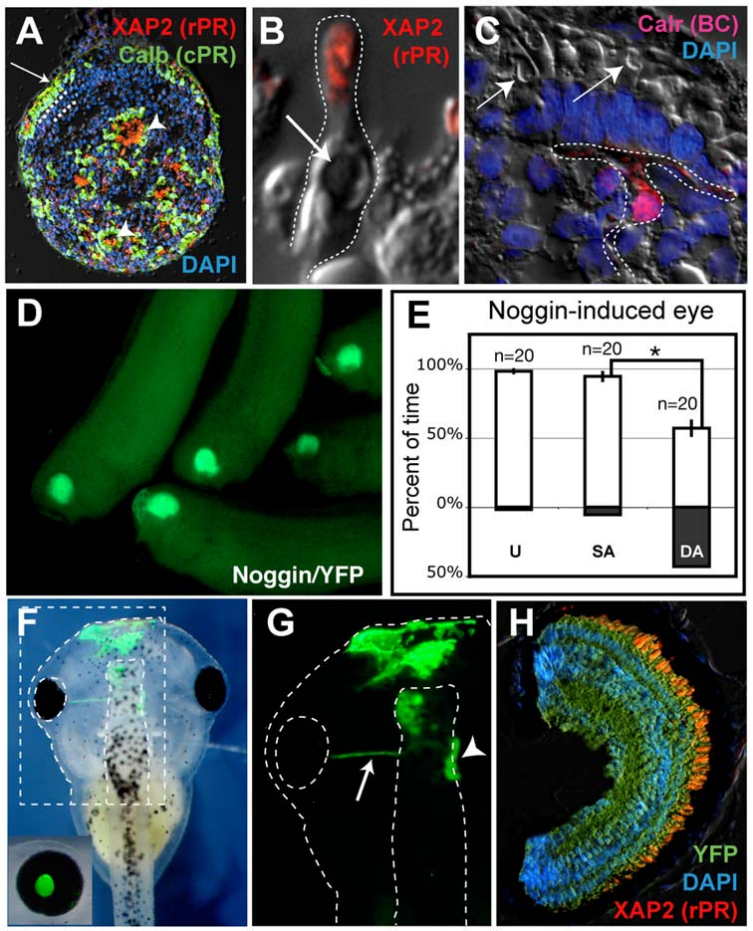
Noggin-treated pluripotent cells generate retinal cells in vitro and form functional eyes in vivo. (A–C) Retinal cells formed in cultured explants treated with Noggin protein. (A) Rosettes (arrowheads) and pseudo outer nuclear layers (arrow) are detected in explants triple stained for nuclei (blue; DAPI), cone (green; Calbindin), and rod photoreceptors (red; XAP2). (B) Cultured cells not only expressed photoreceptor specific markers, but also had morphologies typical of immature photoreceptors including small outer segments and oil droplets (arrow). (C) A Calretinin-expressing cell (red) shows characteristic bipolar cell morphology, extending processes toward a nearby layer of photoreceptors. Arrows indicate the location of representative oil droplets in the photoreceptor layer. (D) Pluripotent cells from YFP transgenics form eyes when treated with Noggin protein and grafted to the eye field of stage 15 wild-type hosts (these stage 32 animals were photographed 1 d posttransplant). (E) The background color preference assay demonstrates that Noggin-induced retinas are functional. No statistical difference was observed prior to (U) and following single axotomy (SA) to blind the control eye. However, following axotomy of the RGC axons from the induced eye (DA), animals showed no background color preference. Graphs show mean±standard error of the mean. * *p* = 0.002 (E). *n* = number of 2-min trials. (F–H) Eye generated using Noggin-treated pluripotent cells (tested in E) was completely donor derived. YFP expression is detected in axons projecting from the induced eye (G, arrow) to the brain (G, arrowhead). YFP expressing cells are also observed in the surface ectoderm and left half of the forebrain (G). (H) Noggin-induced eye stained for YFP (green) throughout demonstrating it was completely donor derived. Induced eye was also stained to detect rod photoreceptors (XAP2, red) and nuclei (DAPI, blue). rPR, rod; cPR, cone photoreceptor; BP, bipolar cell.

We first asked if Noggin-treated *Xenopus* pluripotent cells would differentiate into retinal cells in vitro. Pluripotent cells were cultured in mouse Noggin protein for 5 d and immunocytochemistry was used to detect retinal specific markers. While untreated pluripotent cells never expressed retinal markers (25 explants, npublished data), Noggin-treated explants expressed markers for rod and cone photoreceptors, as well as inner nuclear layer cells (Figure 7A–7C; Table S5). Rods and cones formed both rosettes and pseudo outer nuclear layers similar to those observe in the normal retina, with interspersed rods and cones. Labeled cell classes could also be identified based on their morphology. Oil droplets, outer, and inner segments were observed in cells expressing photoreceptor specific markers (Figure 7B and 7C). Calretinin-expressing (likely bipolar) cells were also observed extending processes toward the peduncles of nearby layered photoreceptors (Figure 7C).

We next transplanted pluripotent cells cultured in Noggin to the neural plate of stage 15 embryos. Every embryo receiving a transplant of Noggin-treated pluripotent cells formed a YFP expressing eye (Figure 7D, N = 38). YFP-only pluripotent cells never formed eyes (unpublished data, N = 31). Embryos were cultured to stage 51, and tested for phototropic behavior. An animal with one Noggin-induced eye and an age-matched wild-type animal spent 98%±0.8% and 96%±3% of swimming time on the white side of the test tank, respectively (Figure 7E and unpublished data). Following single axotomy to blind the right (control) eye, a white color preference was still evident in both animals (Figure 7E and unpublished data). Visual guided behavior was significantly reduced however, when the optic nerve from the second (induced) eye was severed (Noggin-induced eye, 95%±4% to 57%±7%, *p* = 0.002; control, 78%±5% to 52%±5%, *p* = 0.001). We confirmed the induced eye was completely donor derived. YFP-labeled projections were observed and sectioning of the animal tested and confirmed that all cells in every section of the induced eye were YFP-positive (Figure 7F–7H and unpublished data). These results suggest that Noggin, similar to the EFTFs it induces, directs pluripotent cells to a multipotent retinal progenitor lineage. Induced retinal cells differentiate into functional retinal cell classes and form a neural network sufficient for vision.

## Discussion

The induced retinal cells generated here are multipotent, as they differentiate into all retinal cell classes and even express markers for some retinal cell subtypes. We observed stereotypical retinal cell morphology in eyes completely derived from EFTF-expressing pluripotent cells and in mosaic retinas. Lamba and colleagues recently demonstrated that human embryonic stem cells could be directed to differentiate into photoreceptors and respond to a light stimulus as measured by ERG in *crx* ^−/−^ mice (a model for Leber's congenital amaurosis) [5]. This important finding strongly suggests that in vitro generated cells can form functional retinal cells in a nonfunctioning retina. We show that pluripotent cells can generate all retinal cell classes. When transplanted to the anterior neural plate, these cells form eyes and a neural network capable of driving a vision-guided behavior.

Not all donor cells were reprogrammed to a retinal lineage by the EFTFs. Why this might be is unclear. One hypothesis is that a subset of the pluripotent cells was refractory to the effects of the EFTFs. Alternatively, some cells may not have received a sufficient dose or the appropriate relative level of each EFTF. This possibility is intriguing, since subsets of the EFTFs are coordinately expressed in specific regions of the forebrain and developing olfactory placodes [7]. It will be interesting to determine if specific EFTF subsets can fate pluripotent cells to other (nonretinal) neural lineages.

Eye fields and early optic vesicles form eye-like structures with lenses when transplanted ectopically or when cultured in vitro [21]–[26]. Similarly, EFTF-expressing pluripotent cells formed ectopic eye-like structures when transplanted to the embryonic flank. Although induced cells expressed retinal markers and exhibited retinal cell morphology in culture, morphologically normal eyes never formed. These results demonstrate fundamental differences between the induced retinal cells and the embryonic eye field. In isolation, EFTF-expressing pluripotent cells may be unable to recapitulate the complex inductive and morphological changes necessary for complete eye formation in culture. EFTFs may redirect pluripotent cells to a more restricted lineage than isolated eye fields, which may include additional tissues necessary for in vitro eye formation. Our transplantation experiments are consistent with this interpretation. While both eye fields and EFTF-expressing pluripotent cells generated epidermis, forebrain, and olfactory tissue when grafted to the anterior neural plate, transplanted eye fields also formed muscle and head mesenchyme (Figure 4 and unpublished data). A requirement for mesoderm is supported by the work of Asashima, showing that tissue recombinants including lateral marginal zone or the mesoderm inducer Activin A generates muscle as well as *pax6* expressing eye-like structures with lens in vivo as well as in vitro [27],[28]. The presence of lens in these tissue recombinants may also be telling as EFTF-expressing pluripotent cells very infrequently formed lens. The reduced ability of the EFTFs to induce lens in the embryonic flank may be the cause of the abnormal layering we observed, since the lens and retina are dependent on each other for their normal formation.

It is interesting that YFP-expressing pluripotent cells were rarely detected in the lens, yet often present in the cornea since the surface ectoderm forms both the lens and the cornea. All surgeries were performed on stage 15 embryos. At this developmental stage, the presumptive lens ectoderm lies lateral to, and outside the neural plate [29],[30]. Therefore, successful removal of part or the entire eye field would spare the host presumptive lens ectoderm in the majority of the animals. Following neurulation, the externally localized (YFP-negative) lens ectoderm comes to lie over the evaginating optic vesicle, they make contact, the lens placode forms, and together they invaginate to form the optic cup and lens vesicle (reviewed in [31]). We speculate, that once the YFP-negative lens vesicle separates from the surface ectoderm, the cornea forms from the remaining surface ectoderm (YFP-positive) and neural crest-derived mesenchyme [32].

YFP-expressing pluripotent cells did not form neural tissue (retina or brain), but were always observed in the epidermis of tadpoles. This suggests that the transplanted, control cells move from the eye field to the surface ectoderm outside the neural plate. Conversely, EFTF-expressing pluripotent cells (and transplanted eye fields) remained in the anterior neural plate. In *Xenopus*, expression of the EFTFs *pax6*, *rx1*, as well as *otx2* redirect ventral progenitors normally destined to form epidermis into the eye field where they form retina [20]. It will be important to identify the transcriptional targets of the EFTFs and the mechanism(s) responsible for these morphological movements.

Eyes generated from pluripotent cells continued to grow throughout the life of the animals and contained a population of mitotically active cells in the CMZ suggesting the presence of retinal stem cells. During early embryonic development, cells throughout the forming retina are proliferative. Progenitors of the central retina are the first to leave the cell cycle and differentiate into the six neuronal and one glial cell class of the mature retina. In some species (including *Xenopus*), the most peripheral cells of the retina never differentiate but form the retinal stem cells of the CMZ [33],[34]. The Wnt, Shh, BMP, Insulin/IGF, and FGF signaling pathways have all been implicated in establishing and maintaining the adult retinal stem cell niche (reviewed in [34]–[37]). Determining the effect(s) of these extrinsic factors on induced retinal cells and the formation of the CMZ in induced eyes will help define the molecular mechanisms regulating the formation and maintenance of retinal stem cells and the niche in which they reside.

EFTF-expressing pluripotent cells form functional retinal cells and eyes when transplanted to the developing embryo, but can these induced retinal cells also differentiate into all the functional retinal cell classes in a mature normal or damaged retinal environment? Cultured mouse, monkey, and human pluripotent cells can be driven down a retinal lineage as determined by the expression of marker genes, including the EFTFs [2]–[4]. When transplanted to the mouse retina, human ES cells directed to a retinal lineage partially restore light-elicited ERG responses [5]. Noggin is a key component in the cocktail used to bias human ES cells to a retinal lineage, and we found it was also sufficient to direct *Xenopus* pluripotent cells to retinal cell classes and eventually functional eyes. The assays presented here demonstrate a rapid and simple system for testing individual and combinations of intrinsic and extrinsic factors for their ability to direct pluripotent cells to multipotent retinal cells, with the option of testing them functionally. Future studies can now address how to maintain induced retinal cell cultures in a proliferative, multipotent state and drive them to all the retinal cell classes necessary to repair a damaged or degenerating mature retina.

## Materials and Methods

### Microarray Analysis

Eye field (EF) and PNP isolated from regions AB1,2+B2 and HI3,4, respectively of stage 15 embryos (coordinate system described in [38]). LE was isolated from stage 15 lateral flank at A–P coordinates midway between eye field and PNP. Samples were pipetted into microfuge tubes cooled on dry ice to quickly freeze the isolated tissues. RNA from 40 EF, PNP, LE dissected tissues (approximately 250 µm^2^ each) or ten YFP-expressing control or YFP- and EFTF-expressing animal caps (EFTF-expressing pluripotent cells) were used for each chip analysis. Analyses were performed in triplicate on tissue from embryos on three different days from three different females. RNA extraction, probe synthesis and purification, chip hybridization, and scanning were carried out at SUNY-MAC (Syracuse, New York). GeneSpring GX, version 7.3.1 (Agilent Technologies) was used for analysis. Cel file data was preprocessed using RMA method with the average signal intensity normalized to pluripotent cells isolated from Venus YFP RNA injected embryos or stage 15 whole embryos. MIAME-compliant supplementary information is included online at Gene Expression Omnibus (http://www.ncbi.nlm.nih.gov/geo/index.cgi; GSE9173 and GSE9175).

### Culture Conditions and Transplantation of Pluripotent Cells

Capped RNAs coding for *pax6*, *tbx3*, *rx1*, *nr2e1*, *six3*, *six6*, *otx2*, and/or *venus YFP* were generated as previously described and injected into both cells of 2-cell staged transgenic *venus YFP* embryos. Expression of YFP in transgenic embryos is weak until approximately stage 15. Experimental and control embryos were injected with *venus YFP* cRNA, which allowed selection of embryos successfully injected. We collected animal caps at stage 9 [7],[10], also called pluripotent cells here. Tissue was cultured in 0.7× MMR containing 50 µg/ml gentamicin sulphate to the equivalent of stage 15. For Noggin protein-treated caps, stage 9 animal cap explants were cultured with 500 nM mouse Noggin protein (Sigma-Aldrich, catalogue number N6784) in 0.7× MMR and 50 µg/ml gentamicin sulphate and grown to sibling embryo stage 15 for transplantation. A 250-µm^2^ region of LE or the left eye field was removed from stage 15 wild-type host embryos [38] using a Gastromaster. One half of the cultured pluripotent cells was grafted to the wound. For mosaic analysis, 125 µm^2^ (∼1/2) of the left eye field was removed and a size-matched fragment of animal cap was grafted. For Noggin cultured explants, Noggin-treated caps were grown in the same Noggin-containing solution above until sibling tadpoles reached stage 40–41.

### Immunostaining and In Situ Hybridization

Embryos were cultured to the indicated developmental stage and 12-µm, cryostat sections were immunostained as previously described [39]. The following antibodies were used: mouse XAP2 (1∶10; Developmental Studies Hybridoma Bank (DSHB), Iowa City, Iowa, clone 5B9); mouse R5 (1∶5; kindly provided by W.A. Harris, Cambridge University, Cambridge, United Kingdom); rabbit anti-Calbindin (1∶500; VWR, catalog number 80001-624); rabbit anti-GABA (GABA:1∶500; ImmunoStar, catalog number 20094); mouse anti-Islet-1 (1∶100; DSHB clone 39.4D5); rabbit anti-Calretinin (1∶100; Novus Biologicals, catalog number NB200-618); anti-Tyrosine Hydroxylase (TH: 1∶500; ImmunoStar, catalog number 22941); rabbit anti-GFP (1∶750; Invitrogen, catalog number A-11122), mouse anti-GFP (1∶500; Sigma, catalog number G6539). The slides were mounted in solution of FluorSave reagent (VWR), 2%1,4-Diazabicyclo[2.2.2]octane (DABCO: SIGMA) and 10 µg/ml 4′,6-Diamidino-2-phenyindole, dilactate (DAPI: SIGMA). Anti-GABA staining was performed as published [40]. In situ hybridization for hermes was performed as previously described [7],[39].

### Imaging Whole Embryos and Sections

Whole embryos images were captured using a Leica MZ16A fluorescence stereomicroscope with a MicroPublisher 3.3 RTV digital camera (Q-Imaging) and Q-Capture software (V 2.7.1). Sections stained for markers described above were visualized using a Leica DM6000 B upright fluorescence light microscope with motorized Z-focusing. A low-light, high-speed Retiga-SRV camera (Q-Imaging) captured images and sent them to the Volocity Imaging (Improvision Inc. a PerkinElmer Company; V 5.0.3) software. The Volocity software package allowed us to acquire, visualize, deconvolve, and quantitatively measure tissue sections, as well as, retinal cells. Movies were made using this software and exported as QuickTime movies. Eye volumes were calculated by summing the area of every eye section and multiplying by the section thickness (12 µM) as previously described using Volocity Software [19].

### BrdU Incorporation, Staining, and Cell Counts

Tadpoles were allowed to swim in a solution of 5 mM BrdU (Roche Inc.) in 0.1× MMR for 1–4 h at room temperature and sections stained as previously described [39]. Percent of BrdU-labeled cells were determined by dividing their number by the total number of nuclei (DAPI). 50 µm of central retinal sections were compared to the two peripheral regions of the same sections. Peripheral regions were defined as those regions containing cells with an elongated neuroepithelial morphology. In Figure 5O, the number of rod photoreceptor, inner nuclear layer, and retinal ganglion cells were determined by counting the nuclei of cells expressing XAP2, Calretinin, or in the RGC layer, respectively.

### Electrophysiology and Background Color Preference Assay

ERGs were performed as previously described [41]. Vision-based behavioral assay was modified from Moriya et al. [18]. A ½ Gallon Flex-Tank (NASCO) was colored half black and half white (outer tank). Animals were placed in a second clear tank then inside the colored outer tank. The inner tank was rotated 180° following each 2 min trial. Behavior was recorded using a digital camcorder. Ten 2-min trials were run on two consecutive days for a total of 20 trials. A within-subject design, statistical analysis using a Student's *t*-test, paired two-tailed distribution was used. *p*≤0.05 was considered significant. Results shown as mean±standard error of the mean. Sham and unoperated animals behaved similarly (unpublished data).

### Retinal Axotomy

Animals were anesthetized in 0.01% tricaine. The optic nerve was located and a 26-gauge needle and forceps were used to sever and displace the optic nerve from the optic tract. Tadpoles recovered in water containing 50 µg/ml gentamicin. The Committee for the Humane Use of Animals at SUNY Upstate Medical University approved all procedures.

## Supporting Information

Figure S1 An unsupervised hierarchical clustering algorithm groups the transcriptional profile of eye field and EFTF-expressing pluripotent cells together.
(6.44 MB TIF)

Figure S2 EFTF-expressing pluripotent cells differentiate as retinal cells and incorporate seamlessly with host cells to form mosaic retinas.
(5.45 MB TIF)

Figure S3 Example demonstrating how individual cell classes were identified based on morphology and cell class-specific markers.
(1.00 MB TIF)

Figure S4 Z-series movie of the cone photoreceptor derived from EFTF-expressing pluripotent cells in Figure 5D.
(0.66 MB MOV)

Figure S5 Z-series movie of the rod photoreceptor derived from EFTF-expressing pluripotent cells in Figure 5E.
(0.42 MB MOV)

Figure S6 Z-series movie of the horizontal cell derived from EFTF-expressing pluripotent cells in Figure 5F.
(0.68 MB MOV)

Table S1 Transcripts expressed in EFTF-PCs are required for normal eye formation.
(0.06 MB DOC)

Table S2 Table S2. Expression domains and relative expression levels of the PNP and LE genes in Figure 1C.
(0.05 MB DOC)

Table S3 Molecular markers used and the retinal cell types they label.
(0.04 MB DOC)

Table S4 Molecular markers for seven retinal cell classes and peripherally located mitotic cells were detected in flank retinas.
(0.03 MB DOC)

Table S5 Frequency retinal cell classes were detected in primitive ectoderm explants following a five-day culture in media containing noggin protein.
(0.03 MB DOC)

## Abbreviations

BrdU: 5-bromo-2-deoxyuridine
CMZ: ciliary marginal zone
EFTF: eye field transcription factor
ERG: electroretinogram
GABA: gamma-aminobutyric acid
LE: lateral endoderm
N: number of animals or eyes
PNP: posterior neural plate
RPE: retinal pigment epithelium
YFP: yellow fluorescent protein
