# Supplementary figures and images for: Generation of Functional Eyes from Pluripotent Cells
Source: PLoS Biol. 2009 Aug 18;7(8):e1000174. doi: 10.1371/journal.pbio.1000174 (PMC2716519; doi:10.1371/journal.pbio.1000174)

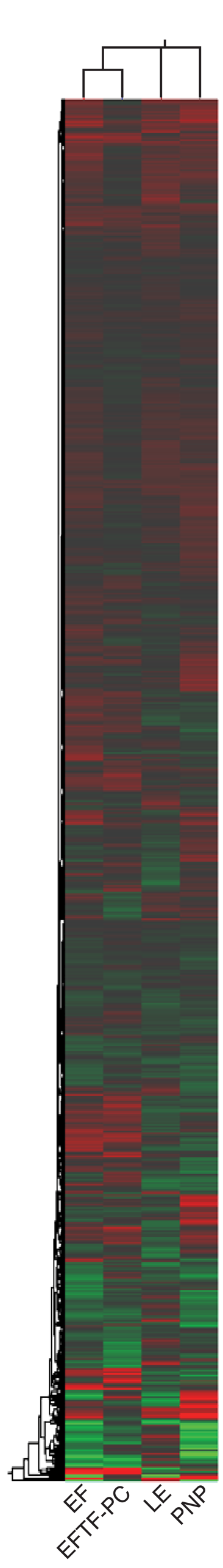

Supplement: Figure S1 — An unsupervised hierarchical clustering algorithm groups the transcriptional profile of eye field and EFTF-expressing pluripotent cells together. (6.44 MB TIF) [file pbio.1000174.s001.tif]

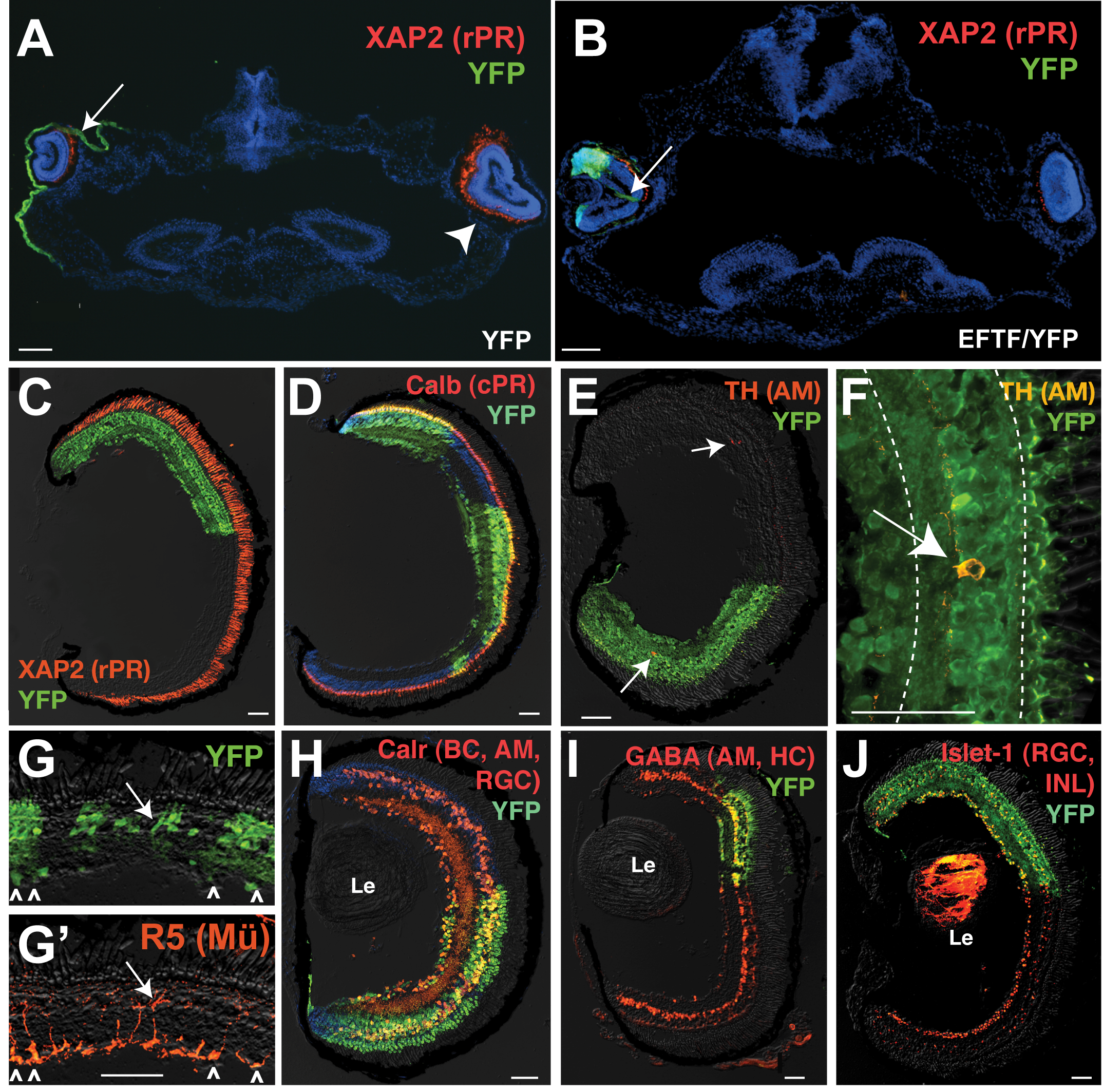

Supplement: Figure S2 — EFTF-expressing pluripotent cells differentiate as retinal cells and incorporate seamlessly with host cells to form mosaic retinas. (5.45 MB TIF) [file pbio.1000174.s002.tif]

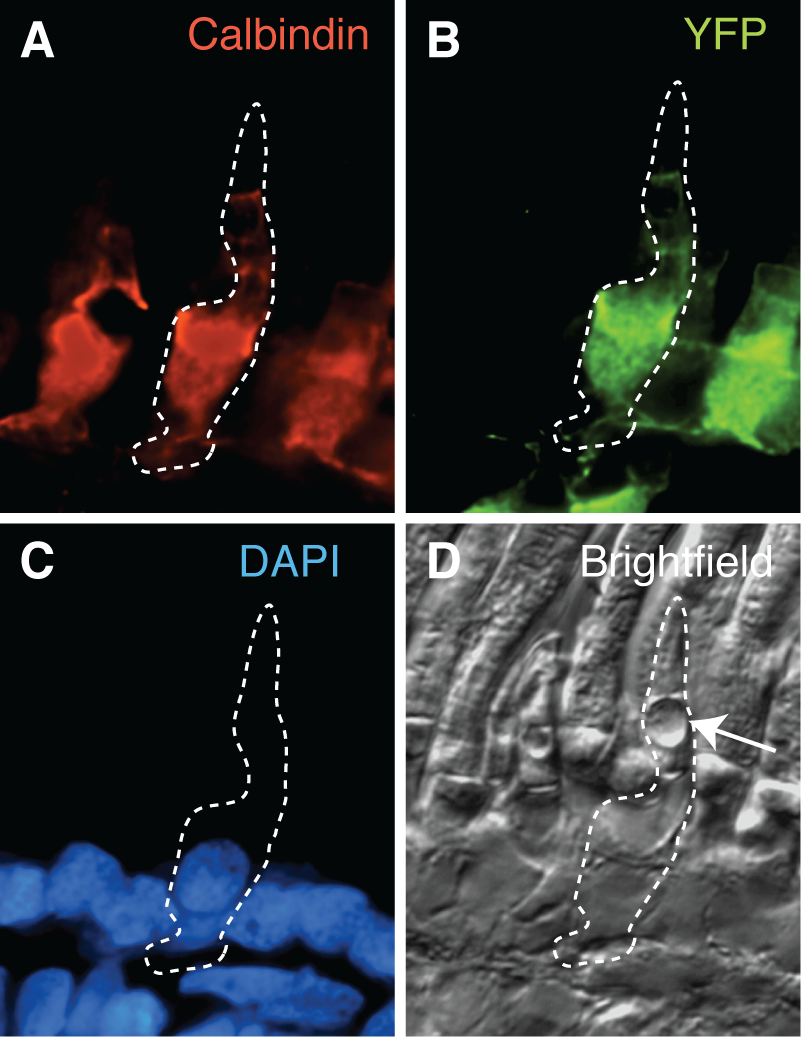

Supplement: Figure S3 — Example demonstrating how individual cell classes were identified based on morphology and cell class-specific markers. (1.00 MB TIF) [file pbio.1000174.s003.tif]
