# Supplementary material for: Generation of Functional Eyes from Pluripotent Cells
Source: PLoS Biol. 2009 Aug 18;7(8):e1000174. doi: 10.1371/journal.pbio.1000174 (PMC2716519; doi:10.1371/journal.pbio.1000174)
Supplement: Table S1 — Transcripts expressed in EFTF-PCs are required for normal eye formation. (0.06 MB DOC) [file pbio.1000174.s007.doc]

| ***Gene*** | **Accession #** | **Ave. fold induction EFTF-PC vs. PC** | **Ave. fold enrichmentEF vs. WE** | **EFTF-PC vs EF p-value** | **Ave. fold enrichmentPNP vs. WE** | **EFTF-PC vs PNP p-value** | **Ave. fold enrichmentLE vs. WE** | **EFTF-PC vs LE p-value** | **Required in** |
| --- | --- | --- | --- | --- | --- | --- | --- | --- | --- |
| *pax6** | AF154558 | 6.67 | 4.44 | 0.109 | 0.25 | **0.001** | 0.20 | **0.003** | H[3], M[4,5], X[6], F[7] |
| *rx/rax** | AF001049 | 10.52 | 5.01 | 0.112 | 0.31 | **0.001** | 0.25 | **0.001** | H[8], M[9], X[10], F[11] |
| *six3** | AF183571 | 4.08 | 4.18 | 0.878 | 0.27 | **0.002** | 0.37 | **0.005** | H[12], M[13], F[14] |
| *lhx2* | BJ087007 | 7.95 | 7.38 | 0.745 | 0.37 | **0.003** | 0.31 | **0.003** | M[15], F[16] |
| *otx2** | AI031473 | 10.62 | 2.69 | **0.0006** | 0.04 | **0.000003** | 0.06 | **0.0002** | H[17], M[18,19], X[20] |
| *sox2* | BJ078362 | 7.63 | 2.08 | **0.003** | 1.39 | **0.001** | 0.43 | **0.001** | H[21], M[22], X[23], F[24] |
| *pax2* | Y10120 | 2.04 | 3.54 | 0.421 | 0.72 | 0.999 | 0.77 | 0.316 | H[25], M[26], F[27] |
| *dkk1* | AF030434 | 2.38 | 3.41 | 0.551 | 1.12 | **0.047** | 0.65 | **0.006** | M[28], X[29], F[30] |
| *otx5/ crx* | BJ056449 | 3.77 | 2.42 | 0.421 | 0.56 | **0.003** | 0.51 | **0.005** | H[31], M[32], X[33], F[34] |
| *Xanf1/hesX1* | X60099 | 13.26 | 3.37 | 0.116 | 0.24 | **0.002** | 0.26 | **0.003** | H[35], M[35], X[36] |
| *vsx1* | BC044049 | 2.83 | 3.79 | 0.565 | 0.50 | **0.039** | 0.76 | 0.108 | H[37], M[38] |
| *hes1* | BC041261 | 2.87 | 3.23 | 0.551 | 0.35 | **0.003** | 0.56 | **0.013** | M[39,40] |
| *Ncad/cdh2* | BG161210 | 6.91 | 2.48 | 0.054 | 0.81 | **0.001** | 0.68 | **0.002** | F[41-43] |
| *otx1* | AY029294 | 4.98 | 5.26 | 0.878 | 0.07 | **0.0004** | 0.37 | **0.001** | M[44,45] |
| *zic2* | AB014461 | 8.98 | 2.34 | 0.059 | 1.31 | 0.831 | 0.72 | **0.030** | X[46], F[47] |
|  |  | **A** | **B** |  | **C** |  | **D** |  |  |

**Table S1. Transcripts expressed in EFTF-PCs are required for normal eye formation.** List of genes that meet *all* the following criteria: 1) induced greater than two-fold in EFTF-expressing pluripotent cells (EFTF-PC) relative to primitive ectoderm, 2) enriched greater than two-fold in eye field (EF) relative to whole embryo (WE) and 3) whose homologs are required for normal eye formation in humans (H), mice (M), *Xenopus* (X) and/or fish (F; medaka or zebrafish). P values were computed using a Student’s unpaired T-test and Benjamini-Hochberg multiple testing correction [48] by comparing the normalized expression value of the EFTF-PC cells versus each of the other tissues (Column A versus B, C or D). Bolded P values (less than 0.05) indicate statistical significance. The microarray probe sets used to detect *pax6, rax, six3 and otx2*, do not hybridize to the injected RNAs, and therefore, the detected increase in expression of these EFTFs results from an increase in transcription from the endogenous genes (asterisk). The EFTF cocktail also included cRNAs encoding *tbx3* (or *ET*), *nr2e1* (or *tailless*) and *six6* (or *optx2*) transcripts.
