# Supplementary material for: Generation of Functional Eyes from Pluripotent Cells
Source: PLoS Biol. 2009 Aug 18;7(8):e1000174. doi: 10.1371/journal.pbio.1000174 (PMC2716519; doi:10.1371/journal.pbio.1000174)
Supplement: Table S2 — Expression domains and relative expression levels of the PNP and LE genes in Figure 1C. (0.05 MB DOC) [file pbio.1000174.s008.doc]

| ***Gene*** | **Accession #** | **Ave. fold induction EFTF-PC vs. PC** | **Ave. fold inductionEF vs. WE** | **EFTF-PC vs EF p-value** | **Ave. fold induction PNP vs. WE** | **EFTF-PC vs PNP p-value** | **Ave. fold inductionLE vs. WE** | **EFTF-PC vs LE p-value** | **Expression domain** |
| --- | --- | --- | --- | --- | --- | --- | --- | --- | --- |
| *hoxc6* | X12500 | 1.01 | 0.44 | **0.004** | 4.67 | **0.001** | 0.34 | **0.006** | PNP: neuro-ectoderm [49,50] |
| *cdx1* | BG016128 | 0.92 | 0.11 | **0.0003** | 3.46 | **0.002** | 0.72 | 0.214 | PNP: all germ layers [51] |
| *cdx4* | U02034 | 0.87 | 0.20 | **0.0002** | 4.51 | **0.000003** | 0.45 | **0.010** | PNP: all germ layers [51] |
| *Xbra* | M77243 | 1.02 | 0.35 | **0.0003** | 8.35 | **0.001** | 0.36 | **0.001** | PNP: mesoderm [52] |
| *derriere* | AF065135 | 0.97 | 0.42 | **0.0003** | 6.12 | **0.001** | 0.47 | **0.001** | PNP: mesoderm [53] |
| *hnf1ß* | BJ080488 | 0.94 | 0.10 | **0.003** | 0.15 | **0.001** | 1.88 | **0.001** | Endoderm [54] |
| *sox17ß* | AJ001742 | 1.09 | 0.11 | **0.0003** | 0.15 | **0.0004** | 1.16 | **0.435** | Endoderm [55] |
| *gata2* | M76564 | 0.38 | 0.18 | 0.090 | 0.14 | **0.025** | 1.61 | **0.046** | Endoderm [56] |
| *gata3* | M76565 | 0.86 | 0.26 | **0.001** | 0.17 | **0.001** | 1.79 | 0.154 | Endoderm [57] |
| *gata5* | L13702 | 0.98 | 0.34 | **0.001** | 0.41 | **0.0005** | 2.35 | **0.001** | Endoderm [58] |
| *gata6* | BJ090294 | 0.76 | 0.08 | **0.0003** | 0.02 | **0.00001** | 1.46 | **0.010** | Endoderm [59] |
|  |  | **A** | **B** |  | **C** |  | **D** |  |  |

**Table S2.** **Expression domains and relative expression levels of the PNP and LE genes in Fig. 1 *C*.** P values were computed as described in Table S1. Bolded P values indicate statistically significant differences (P < 0.05) in gene induction between normalized EFTF-PC (Column A) and each of the three normalized tissue data sets indicated (Columns B, C or D).
