# Supplementary material for: Generation of Functional Eyes from Pluripotent Cells
Source: PLoS Biol. 2009 Aug 18;7(8):e1000174. doi: 10.1371/journal.pbio.1000174 (PMC2716519; doi:10.1371/journal.pbio.1000174)
Supplement: Table S3 — Molecular markers used and the retinal cell types they label. (0.04 MB DOC) [file pbio.1000174.s009.doc]

|  | RGC | AM | BC | HC | Mü | cPR | rPR | Ref |
| --- | --- | --- | --- | --- | --- | --- | --- | --- |
| Islet 1* | **X** |  |  |  |  |  |  | [60] |
| hermes | **X** |  |  |  |  |  |  | [61] |
| Calretinin | **X** (subset) | **X** (GABAergic & Serotonin) | **X** |  |  |  |  | [62] |
| GABA |  | **X** (GABAergic subset) |  | **X** |  |  |  | [63] |
| TH |  | **X** (Dopaminergic subset) |  |  |  |  |  | [64] |
| R5 |  |  |  |  | **X** |  |  | [65] |
| Calbindin |  |  |  |  |  | **X** |  | [33] |
| XAP2 |  |  |  |  |  |  | **X** | [66] |

**Table S3. Molecular markers used and the retinal cell types they label.** Cell types are retinal ganglion cells (RGC), amacrine (AM), bipolar (BC), horizontal (HC), Müller glia (Mü), cone photoreceptor (cPR), rod photoreceptor (rPR). Antibodies include mouse XAP2 (1:10; Developmental Studies Hybridoma Bank, Iowa City, IA (DSHB) clone 5B9); mouse R5 (1:5; kindly provided by W.A. Harris, Cambridge University, Cambridge, UK); rabbit anti-Calbindin (1:500; VWR, West Chester, PA); rabbit anti-GABA, gamma-aminobutyric acid (1:500; ImmunoStar, Hudson, WI, Cat#: 20094); mouse anti-Islet 1 (1:100; DSHB clone 39.4D5), anti-TH, Tyrosine Hydroxylase (1:500; ImmunoStar; Cat#: 22941); rabbit anti-Calretinin (1:100; Novus Biologicals, Littleton, CO, Cat#: NB200-618). * Because Islet-1 labels inner nuclear layer cells in addition to RGCs at late developmental stages, we also used *in situ* hybridization to detect *hermes* transcription, which is restricted to the RGCs in the retina.
