# Supplementary material for: Generation of Functional Eyes from Pluripotent Cells
Source: PLoS Biol. 2009 Aug 18;7(8):e1000174. doi: 10.1371/journal.pbio.1000174 (PMC2716519; doi:10.1371/journal.pbio.1000174)
Supplement: Table S4 — Molecular markers for seven retinal cell classes and peripherally located mitotic cells were detected in flank retinas. (0.03 MB DOC) [file pbio.1000174.s010.doc]

|  | **Tri-layered (n = 31)** | **Abnormally layered (n = 97)** |
| --- | --- | --- |
| RGC (Islet-1*) | 100% (n = 7) | 92% (n = 24) |
| RGC (*hermes*) | 100% (n = 5) | 93% (n = 15) |
| Amacrine (TH) | 88% (n = 8) | 79% (n = 28) |
| Müller glia (R5) | 100% (n = 9) | 100% (n = 33) |
| Rod PR (XAP2) | 100% (n = 18) | 84% (n = 70) |
| Cone PR (Calbindin) | 100% (n = 9) | 90% (n = 58) |
| HC & Amacrine subset (GABA) | 100% (n = 5) | 94% (n = 18) |
| BC & AM, RGC subsets (Calretinin) | 100% (n = 3) | 100% (n = 14) |
| Mitotic cells in periphery (BrdU) | 100% (n = 16) | 100% (n = 59) |

**Table S4. Molecular markers for seven retinal cell classes and peripherally located mitotic cells were detected in flank retinas.** Flank retinas formed either a stereotypical tri-layered retinal structure or were abnormally layered. In both cases, however, the expression of cell type specific markers was observed in cells aligned into rows. Each flank retina was stained for two or more retinal markers. Consequently, the total number of stained retinal sections is greater than the total flank retina number. * Islet-1 specifically labels RGCs until at least stage 33/34. By stage 40, Islet-1 also labels inner nuclear layer cells. Since it is not possible to determine the developmental stage of flank retinas, the cells labeled with Islet-1 are either RGCs or a combination of RGCs and INL cells. Horizontal cell (HC); bipolar cell (BC); amacrine cell (AM); retinal ganglion cell (RGC).
