# Supplementary material for: Generation of Functional Eyes from Pluripotent Cells
Source: PLoS Biol. 2009 Aug 18;7(8):e1000174. doi: 10.1371/journal.pbio.1000174 (PMC2716519; doi:10.1371/journal.pbio.1000174)
Supplement: Table S5 — Frequency retinal cell classes were detected in primitive ectoderm explants following a five-day culture in media containing noggin protein. (0.03 MB DOC) [file pbio.1000174.s011.doc]

| **Cell class**  (marker) | **Rod PR** (XAP2) | **Cone PR** (calbindin) | **BC, AM, RGC**  (calretinin) |
| --- | --- | --- | --- |
| Noggin-treated  explants | 33%  (n = 46) | 33%  (n = 46) | 43%  (n = 46) |

**Table S5. Frequency retinal cell classes were detected in primitive ectoderm explants following a five-day culture in media containing noggin protein.** Bipolar cell (BC); amacrine cell (AM); retinal ganglion cell (RGC).
